# Long non-coding RNA Gm2199 rescues liver injury and promotes hepatocyte proliferation through the upregulation of ERK1/2

**DOI:** 10.1038/s41419-018-0595-9

**Published:** 2018-05-22

**Authors:** Qiang Gao, Yunyan Gu, Yanan Jiang, Li Fan, Zixiang Wei, Haobin Jin, Xirui Yang, Lijuan Wang, Xuguang Li, Sheng Tai, Baofeng Yang, Yan Liu

**Affiliations:** 1 Department of Physiology, Harbin Medical University, 150081 Harbin, China; 2 College of Bioinformatics Science and Technology, Harbin Medical University, 150081 Harbin, China; 3 Department of Pharmacology (State-Province Key Laboratories of Biomedicine and Pharmaceutics), Harbin Medical University, 150081 Harbin, China; 4 Department of Materia Medica (State Key Laboratory of Bioactive Substance and Function of Natural Medicines), Chinese Academy of Medical Sciences and Peking Union Medical College, 100050 Beijing, China; 5 Department of General Surgery, The Second Affiliated Hospital of Harbin Medical University, 150001 Harbin, China

## Abstract

Long non-coding RNAs (lncRNAs) are a new class of regulators of various human diseases. This study was designed to explore the potential role of lncRNAs in experimental hepatic damage. In vivo hepatic damage in mice and in vitro hepatocyte damage in AML12 and NCTC1469 cells were induced by carbon tetrachloride (CCl_4_) treatments. Expression profiles of lncRNAs and mRNAs were analyzed by microarray. Bioinformatics analyses were conducted to predict the potential functions of differentially expressed lncRNAs with respect to hepatic damage. Overexpression of lncRNA Gm2199 was achieved by transfection of the pEGFP-N1-Gm2199 plasmid in vitro and adeno-associated virus-Gm2199 in vivo. Cell proliferation and viability was detected by cell counting kit-8 and 5-ethynyl-2′-deoxyuridine assay. Protein and mRNA expressions of extracellular signal-regulated kinase-1/2 (ERK1/2) were detected by western blot and quantitative real-time reverse-transcription PCR (qRT-PCR). Microarray analysis identified 190 and 148 significantly differentially expressed lncRNAs and mRNAs, respectively. The analyses of lncRNA-mRNA co-expression and lncRNA-biological process networks unraveled potential roles of the differentially expressed lncRNAs including Gm2199 in the pathophysiological processes leading to hepatic damage. Gm2199 was downregulated in both damaged livers and hepatocyte lines. Overexpression of Gm2199 restored the reduced proliferation of damaged hepatocyte lines and increased the expression of ERK1/2. Overexpression of Gm2199 also promoted the proliferation and viability of normal hepatocyte lines and increased the level of p-ERK1/2. Overexpression of Gm2199 in vivo also protected mouse liver injury induced by CCl_4_, evidenced by more proliferating hepatocytes, less serum alanine aminotransferase, less serum aspartate aminotransferase, and decreased hepatic hydroxyproline. The ability of Gm2199 to maintain hepatic proliferation capacity indicates it as a novel anti-liver damage lncRNA.

## Introduction

Hepatic damage casts adverse influence on human health with a high morbidity worldwide^1^. It can be caused by diverse toxic substance, for example, alcohol and reactive oxygen species^2,3^. Persistent hepatic damage can lead to large-scale hepatocyte damages accompanied by severe fibrosis and irreversible cirrhosis that often causes death^4,5^. Much factors participating hepatic damage are still unclear, which hinders treatments. Therefore, better understanding of the factors involved in hepatic damage is of paramount importance for the treatment of the associated liver diseases.

Hepatocytes play the roles of metabolizing quantities of substances in liver^6^, which are easy to be attacked by toxin followed by inflammation or necrosis^7,8^. Hepatocytes possess high abilities of fission and proliferation, which are able to increase proliferation to compensate the lost ones so as to maintain hepatic functions. Although hepatic stellate cells (HSCs) are also able to proliferate to compensate damaged hepatocytes, they will produce much extracellular matrix (ECM) surrounding the hepatocytes to inhibit proliferation. Hence, promoting the proliferation of hepatocytes and inhibiting their death under hepatic damages have been considered important therapeutic strategies^5,9^.

Long non-coding RNAs (lncRNAs), a class of ncRNAs ranging from 200 bp to >100 kb, have been recognized as critical regulatory molecules for many cellular functions and pathological processes^10,11^. LncRNAs regulate gene expression and associated cellular functions at pre-, post-, or transcriptional level to participate disease development^12^. Particularly, their potential roles in hepatic carcinoma (HCC) and liver fibrosis have been appreciated. LncRNA HOTAIR, HULC, uc003wbd, and some others were found highly expressed in patients with HCC or hepatic B virus, with the potential as new biomarkers for diagnosis^13–15^. LncRNA MEG3 is downregulated in HCC and liver fibrosis^16^. However, most of the investigations have been being focused on the interactions between lncRNAs and HSCs or HCCs^17,18^, and systematic studies on the relationship between lncRNAs and hepatic damage are still lacking.

Extracellular signal-regulated kinase-1/2 (ERK1/2) are widely expressed in various cells including cardiomyocytes, neurons, and hepatocytes^19–21^. They are directly activated by phosphorylation of mitogen-activated protein kinase (MAPK) to promote cellular differentiation, proliferation, and survival^22,23^. Many factors have been proven to promote the activation or the expression of ERK1/2 to promote cellular proliferation^24–26^. The overexpression of lncRNA UCA1 is able to activate the ERK1/2 pathway to promote the proliferation of HCC cells^27^. Additionally, the activation of hepatocyte ERK1/2 during liver injury and hepatectomy was proved to increase hepatocyte proliferation^28,29^. Therefore, the way to increase hepatic ERK1/2 activation and expression seem to be an active way to promote liver regeneration from damage^30^.

Herein, we aimed to acquire expression profiles of lncRNAs and messenger RNAs (mRNAs) in a mouse model of liver damage to identify the deregulated lncRNAs under this pathological setting. The second objective was to obtain some preliminary information on the potential roles of the deregulated lncRNAs in liver damages based upon theoretical analyses. Finally, we also made efforts to understand the functionality of a selected lncRNA named Gm2199 in promoting hepatocyte proliferation with experimental approaches, which was proved to increase the expression of ERK1/2 in damaged hepatocyte.

## Materials and methods

### Animals

Four-week-old male ICR mice (*n* = 30, 20–30 g) were purchased from the experimental Animal Center of Harbin Medical University, which all received humane care under identical conditions in a clean environment with a 12:12-h light/dark cycle and common mouse food and water at will. The mice were accommodated to the new environment for 1 week prior to any treatment. All animal studies were conducted in accordance with the Guidelines for the Care and Use of Laboratory Animals set by the US National Institutes of Health (NIH publication no. 85–23, revised 1996). Meanwhile, all animal experimental protocols were pre-approved by the Experimental Animal Ethic Committee of the Harbin Medical University, China (Animal Experimental Ethical Inspection protocol no. 2009104).

For microarray detection, mice were randomly divided into control group and tetrachloride (CCl_4_) group (*n* = 7 for each group). For confirming the protecting role of Gm2199 in liver injury, mice were randomly divided into control group, CCl_4_ group, CCl_4_ + AAV-Gm2199 group and CCl_4_ + AAV-NC group (*n* = 4 for each group).

### In vivo mouse model of hepatic damage

Mice were injected intraperitoneally with a 20% solution of CCl_4_ in corn oil as a CCl_4_ group or pure corn oil as a control group. The mice received 2.5 mL CCl_4_ solution or oil per kilogram of body weight two times a week for 3 weeks. Mortality and weight were monitored weekly. The animals were killed 48 h after the final injection of CCl_4_^31^, and blood and livers were collected immediately and stored properly for subsequent examinations.

### In vitro model of hepatic damage in mouse hepatocytes

To establish an in vitro model of hepatic damage, mouse hepatic cell line, AML12 (SCSP-550, Stem Cell Bank of Chinese Academy of Sciences, China) and NCTC1469 (NCTC 1469, Tongpai Shanghai Biological Technology Co., China) were cultured in a 1:1 mixture of Dulbecco’s modified Eagle’s medium and Ham’s F12 medium (Sj204, Hyclone, USA) containing ITS Liquid Media Supplement 1× (I3146, Sigma, USA), dexamethasone (40 ng/mL, D4902, Sigma, USA), and 10% fetal bovine serum (Fsp500, Excell Bio, China) at 37 °C with 5% CO_2_. After 24 h, the medium was replaced by fresh medium without serum. The fresh medium contained 6–15 mmol/L CCl_4_ dissolved in DMSO at a volume ratio of 1:1. As a control, cells were cultured in the same fresh medium without serum, which contained the same volume of DMSO as the CCl_4_ group. All the cells were cultured for additional 24 h before assessments.

### Liver histopathological analysis

The livers removed from the mice were weighted, and then fixed partially in 4% paraformaldehyde for 48 h at room temperature. Having been paraffin-embedded and sectioned into 5 μm pieces, the liver sections were stained with hematoxylin and eosin (H&E) and Masson’s trichrome for microscopic examination. Morphology and pathology were analyzed by two independent pathologists under blinded conditions.

### The measurement of alanine aminotransferase and aspartate transaminase in serum

The blood collected from right ventricle was stored at room temperature for 4 h and centrifuged at a speed of 3000 r.p.m. for 10 min. Then, the supernatant was separated. The content of serum alanine aminotransferase (ALT) and aspartate transaminase (AST) were detected following the manufacturer’s protocol of respective assay kits (C009-2 and C010-2, Nanjing Jiancheng Bioengineering Institute, Nanjing, China). The OD value at 510 nm of each well was detected by ELIASA (Infinite 200 PRO, Tecan), and the absolute OD values were obtained from the difference between test well and its paired control well. The unit of ALT or AST expressed as U/L was transformed from the absolute optical density (OD) of each sample according to the standard curve.

### The measurement of liver hydroxyproline

After washed with sterile phosphate-buffered saline (PBS), fresh liver samples were cut into 80–100 mg pieces, dried, weighted precisely, and digested in 1 mL hydrolysate of hydroxyproline (HYP) assay kit (A030-2, Jiancheng Bioengineering Institute, Nanjing, China) at 95 °C for 20 min. After adjusting pH to 6.0–6.8, ddH_2_O was added into the hydrolysate of each sample to 10 mL. Then, 4 mL of diluted hydrolysate was filled with 25 mg active carbon, followed by mixing and centrifugation at 3500 r.p.m. for 10 min. One mililiter of the supernatant was dissolved in ddH_2_O or 5 µg/mL standard applied solution with reaction regents of the HYP assay kit followed by incubating at room temperature and 60 °C for 10 to 15 min. Then the reaction liquids are centrifuged at 3500 r.p.m. for 10 min following cooling. Two-hundred microliter of the supernatant of each sample was dissolved in ddH_2_O or 5 µg/mL standard applied solution in a well of a 96-well microplate, followed by measurement of absorbance at 550 nm. The content of liver HYP was expressed as μg/mg wet liver weight.

### Quantitative real-time reverse-transcription PCR

Total RNA was extracted from liver tissues using Trizol (15596-026, Invitrogen, Carlsbad, CA) according to manufacturer’s instructions. RNA quantity and quality were measured by NanoDrop ND-1000. Complementray DNAs (cDNAs) were synthesized using 0.5 mg of total RNA, oligo(dT)12–18 primers, and a ReverTra Ace quantitative real-time reverse-transcription PCR (qRT-PCR) RT kit (FSQ-101, Toyobo, Osaka, Japan) following manufacturer’s protocol. Gene expression was detected by qRT-PCR using the cDNAs, THUNDERBIRD SYBR qRT-PCR mix reagents (QPS-201, Toyobo, Osaka, Japan), and gene-specific oligonucleotide primers (listed in Supplementary Table S1) with an ABI 7500 fast real-time PCR system (Applied Bio-systems, USA). The expression level of GAPDH was used to normalize the relative abundance of RNAs. Significance was determined by taking the average of the GAPDH-normalized 2^−ΔΔCT^ values.

### Microarray analysis of lncRNAs and mRNAs

Arraystar mouse lncRNA Microarray V3.0 designed for the global profiling of mouse lncRNAs and protein-coding transcripts was used for detecting lncRNAs and mRNAs.

RNA quantity and quality were measured by NanoDrop ND-1000, and RNA integrity was assessed by standard denaturing agarose gel electrophoresis. Sample labeling and array hybridization were performed according to the Agilent One-Color Microarray-Based Gene Expression Analysis protocol (Agilent Technology) with minor modifications. Briefly, mRNAs were purified from total RNA samples after removal of rRNA (mRNA-ONLY™ Eukaryotic mRNA Isolation Kit, Epicentre). Then, each sample was amplified and transcribed into fluorescent cRNA along the entire length of the transcripts without 3′ bias utilizing a random priming method (Arraystar Flash RNA Labeling Kit, Arraystar). The labeled cRNAs were purified by RNeasy Mini Kit (Qiagen). The concentration and specific activity of the labeled cRNAs (pmol Cy3/μg cRNA) were measured by NanoDrop ND-1000. One microgram of each labeled cRNA was fragmented by adding 5 μL 10× Blocking Agent and 1 μL of 25× fragmentation buffer, and the mixture was heated at 60 °C for 30 min. Finally, 25 μL 2× GE hybridization buffer was added to the samples to dilute the labeled cRNA. Fifty microliters of hybridization solution was dispensed into the gasket slide and assembled onto the lncRNA expression microarray slide. The slides were incubated for 17 h at 65 °C in an Agilent Hybridization Oven. The hybridized arrays were washed, fixed, and scanned using the Agilent DNA Microarray Scanner (part number G2505C).

Agilent Feature Extraction software (version 11.0.1.1) was used to analyze the acquired array images. Quantile normalization and subsequent data processing were performed using the GeneSpring GX v12.1 software package (Agilent Technologies). After quantile normalization of the raw data, the lncRNAs and mRNAs that have flags in Present or Marginal (“All Targets Value”) from at least 5 out of 14 samples were chosen for further data analysis. When multiple probes were mapped to the same gene ID, the mean value was used to represent the expression value of the single gene. Differentially expressed lncRNAs and mRNAs between the two samples were identified through fold change (FC) filtering. A two-sample *t* test was carried out to detect differentially expressed lncRNAs and mRNAs, respectively. Differentially expressed lncRNAs and mRNAs with statistical significance between the two groups were identified through *P* value/false discovery rate (FDR) filtering. The *P* values were adjusted by the Benjamin and Hochberg correction procedure to account for multiple tests.

### Bioinformatics analyses of microarray data

Hierarchical clustering of lncRNA expression values was performed with R software using the distance metric as one minus person correlation coefficient and average linkage. The co-expression network between lncRNAs and genes was built according to the Pearson’s correlation coefficients of expression values of lncRNAs and mRNAs. Only the lncRNA-mRNA with |r| > 0.9 and FDR <0.05 were set as significant correlation edges in the co-expression network. We performed functional enrichment using the biological process terms in Gene Ontology (GO) database. The biological process terms annotated with more than three genes and <500 genes were included in our analysis. The hypergeometric distribution model was used to test whether the biological process terms were enriched with the differentially expressed mRNAs. For one lncRNA, if the targets of this lncRNA were significantly overlapped with the targets annotated in one biological process term, we connected the lncRNA and the biological process term in the lncRNA-biological process network with *P* value <0.05 calculated by hypergeometric model. The Cytoscape software (http://www.cytoscape.org/) was used to present the lncRNA-gene co-expression network and lncRNA-biological process network. Pathway analysis was performed using the standard enrichment computation method.

### Transfection of lncRNA Gm2199 into cultured hepatocytes

For overexpression of Gm2199, plasmid pEGFP-N1 (BglIII/PstI, N0253t, Shanghai Generay Biotech, China) was conjugated with full-length Gm2199 DNA (pEGFP-N1‒Gm2199). A pEGFP-N1 conjugated with noting (pEGFP-N1 vector) was kept as a negative control. Cells were incubated in a six-well plate at a density of 1 × 10^5^ per well and a 96-well plate at a density of 4 × 10^3^ per well before transfection. After 24 h, pEGFP-N1‒Gm2199 or pEGFP-N1 vector was transfected into cells using X-treme GENE HP DNA Transfection Reagent (11749800, Roche, Switzerland). All cells were collected after 24 h for the assessments.

### Lactate dehydrogenase cytotoxicity assay

The lactate dehydrogenase (LDH) assay was performed using the LDH Cytotoxicity Assay Kit (A020-2, Nanjing Jiancheng Bioengineering Institute, Nanjing, China) according to the manufacturer’s protocol. The amount of LDH released by cells can be used as an index of cellular damage. The kit contains WST (4-[3-(4-iodophenyl)-2-(4-nitrophenyl)-2H-5-tetrazolio]-1,3-benzene disulfonate) reagent for detection of LDH released from the damaged cells. Reacting with WST, LDH oxidizes lactate to generate NADH, which can be quantified at 450 nm optical density by a ELIASA (Infinite 200 PRO, Tecan).

### CCK-8 assay

Cell proliferation was measured by CCK-8 assay. Exponentially growing cells were resuspended in 200 μL of cell culture medium, seeded at a density of 4 × 10^3^ cells per well in 96-well plates, and incubated overnight for 24 h at 37 °C in a CO_2_ incubator. Cells were added to 10 μL of CCK-8 reagent (Bs350a, Biosharp, China) according to the instructions. The OD value was measured at a wavelength of 450 nm using an ELIASA microplate. Each group was set up in three wells, and each measurement was repeated at least three times.

### Edu staining for hepatocyte in vitro and in vivo

The proliferation of hepatocyte in vitro and in vivo was detected with Cell-Light™ EdU Apollo^®^ 488 In Vitro Imaging Kit (lot no. C10310-3, RiboBio, Guangzhou, China) according to the manufacturer’s protocol. For in vitro detection, hepatocytes were cultivated onto slide at the density of 4 × 10^4^. After different interferences, the cells were stained by 50 μmol/L Edu for 2 h and then fixed with 4% paraformaldehyde in PBS for 30 min. After incubated with 2 mg/mL glycine for 5 min, the cells were incubated with 0.5% Triton X-100 in PBS for 10 min. The cells were then incubated with an Apollo^®^ reaction cocktail containing Apollo^®^ reaction buffer, catalytic agent, fluorochrome, and annexing agent buffer for 30 min while protected from light. The cells were washed once >0.5% Triton X-100 in PBS. For subsequent DNA staining, cells were incubated with 5 μg/mL Hoechst 33342 for 30 min in dark room, and then washed twice with PBS. All steps were carried out at room temperature. To detect proliferating hepatocytes in vivo, mice were received Edu from tail vein 2 h before killing. Then livers were collected for 5 μm frozen sections. The sections were fixed with 4 ℃ acetone for 30 min and washed with 0.5% Triton X-100 in PBS for 10 min at room temperature. Then the sections were incubated with Apollo^®^ reaction cocktail for 30 min while protected from light and the following steps were as described as in vitro detection.

### The overexpression of lncRNA in mouse livers

To overexpress lncRNA Gm2199 in mouse livers, AAV-Gm2199 and AAV-NC were generated from Hanbio, Shanghai, China (contract number HH20170322LLN-AAV01V4). The titration of the virus was 1.4 × 10^12^ v.g./mL, and 100 μL of the virus was injected for one mouse through tail vein at 1 week before the injection of CCl_4_.

### Western blot analysis

Whole cellular protein was extracted from hepatocyte line after incubation with or without CCl_4_. Ten microgram of the protein was separated by 12% SDS-PAGE and transferred onto PVDF membranes. The membranes were blocked with TBST contained with 5% nonfat milk for 2 h at room temperature, and then were incubated with rabbit polyclonal antibody ERK1/2, p-ERK1/2, and GAPDH (4370S, 4695S, and 14C10, Cell Signaling Technology, USA) overnight at 4℃. After washed, the membranes were incubated with HRP-conjugated rabat-anti-goat IgG for 2 h at room temperature. The signal of immuno-band was detected by an ECL reagent (29050, Engreen Biosystem Co., China).

### Statistical analysis

All data, except pathological findings, are presented as mean ± SEM. The significant differences in the data between two groups were determined by Student’s *t* test and among the groups by one-way analysis of variance for equality of variances using SPSS 17.0 (IBM, USA). A *P* value <0.05 was considered statistically significant.

## Results

### The establishment of in vivo and in vitro mouse hepatic damage models

**Fig. 1:**
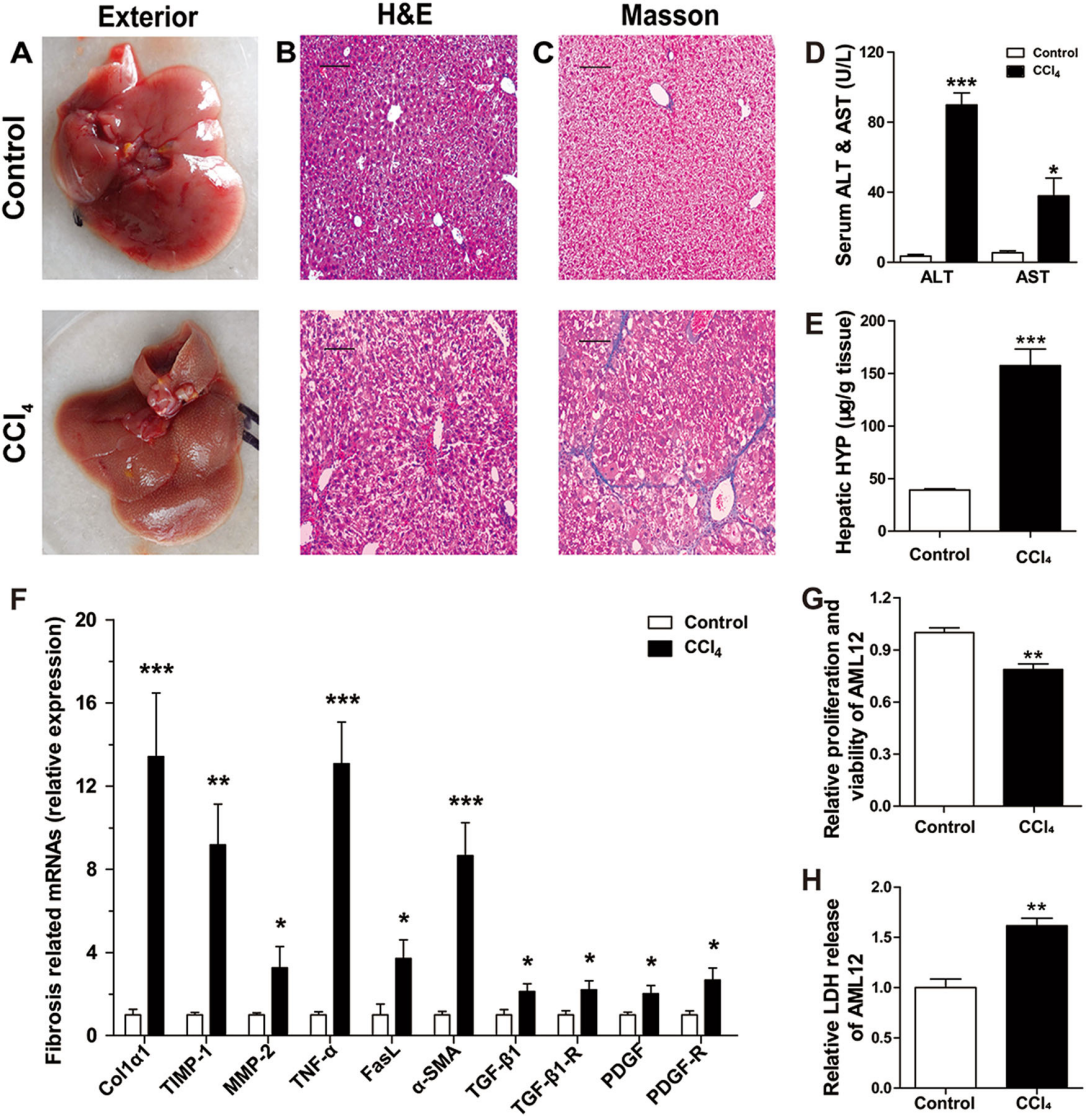
Characterization of mouse model of in vivo hepatic damage induced by injection of CCl_4_ for 3 weeks, and of in vitro hepatocyte damage induced by CCl_4_ for 24 h. **a** Comparison of the exterior appearance of livers between the CCl_4_ and control groups; (**b**) comparison of H&E staining of liver section between the CCl_4_ and control groups showing the differences of hepatic architecture; (**c**) comparison of Masson trichrome staining of liver section between the CCl_4_ and control groups showing the differences of hepatic fibrosis; the scale bars represent 10 μm. **d** Comparison of the contents of serum ALT and AST between the CCl_4_ and control groups. *N* = 7 for each group. **e** Comparison of the contents of hepatic HYP between the CCl_4_ and control groups. *N* = 7 for each group. **f** Alterations of expression levels of the genes related to hepatic damages in CCl_4_-treated mice compared with the control animals, as determined by qRT-PCR. The genes detected included Col1α1, α-SMA, TIMP-1, MMP-2, TNF-α, and TGF-β1. *N* = 7 for each group. **g** The relative cellular proliferation and viability was measured by CCK-8 assay in normal control and damaged AML12 induced by CCl_4_. *N* = 3 for each group. **h** Relative LDH release in normal control and CCl_4_-treated AML12. *N* = 3 for each group. All of the data were expressed as mean ± SEM. *P < 0.05, **P < 0.01, and ***P < 0.001 vs. control

Compared with control group, the livers from CCl_4_ group had exterior appearance of toughness and roughness with numerous white small nodules (Fig. 1a). H&E and Masson’s trichrome staining revealed that the control livers had normal architecture with little fibrous portal expansion (Fig. 1b, c). Whereas, CCl_4_-treated livers exhibited massive fatty changes, gross bridge necrosis, and broad infiltration of neutrophils and lymphocytes (Fig. 1b). CCl_4_-treated livers also showed significant pericentral fibrosis with extensive blue-stained fibers (Fig. 1c). Serum ALT and AST are the most commonly used biochemical markers of hepatic damages^32^. They were respectively increased markedly by 25.4-fold (*P* < 0.001) and 7.0-fold (*P* < 0.05) in CCl_4_ group compared to control group (Fig. 1d). HYP, known to exist in collagens, can reflects the content of organ collagens. In our research, the content of HYP in CCl_4_-treated livers was elevated by 2.44-fold (*P* < 0.001) compared with the control (Fig. 1e). These results indicated that the CCl_4_-treated livers had progressed into the stage of liver fibrosis representing a severe degree of hepatic damage. Furthermore, the expressions of six mRNAs were significantly increased from 2.12-fold–13.42-fold with *P* < 0.001 to 0.05 in CCl_4_ group compared with the control (Fig. 1f). The mRNAs include Col1α1 (collagen type I alpha 1), an indicator of fibrosis, α-SMA (α smooth muscle actin), a marker of HSC activation, TIMP-1 (tissue inhibitor of metalloproteinase 1) and MMP-2 (matrix metallopeptidase 2) involved in ECM disorder, TNF-α (tumor necrosis factor alpha) correlated with hepatocyte apoptosis and TGF-β1 (transforming growth factor beta 1) related with the proliferation of HSCs. They have been reported to be correlated with hepatic damage and fibrosis^33^. Additionally, damaged hepatocyte line, AML12, was also induced by CCl_4_ treatment in vitro. The cellular proliferation and LDH release was significantly downregulated and upregulated respectively in damaged AML12, compared with control (Fig. 1g, h). These data indicated the successful development of an in vivo model of liver damage and an in vitro model of hepatocyte damage for our subsequent experimental investigations.

### The expression profiles of lncRNAs and mRNAs in hepatic damage

**Fig. 2:**
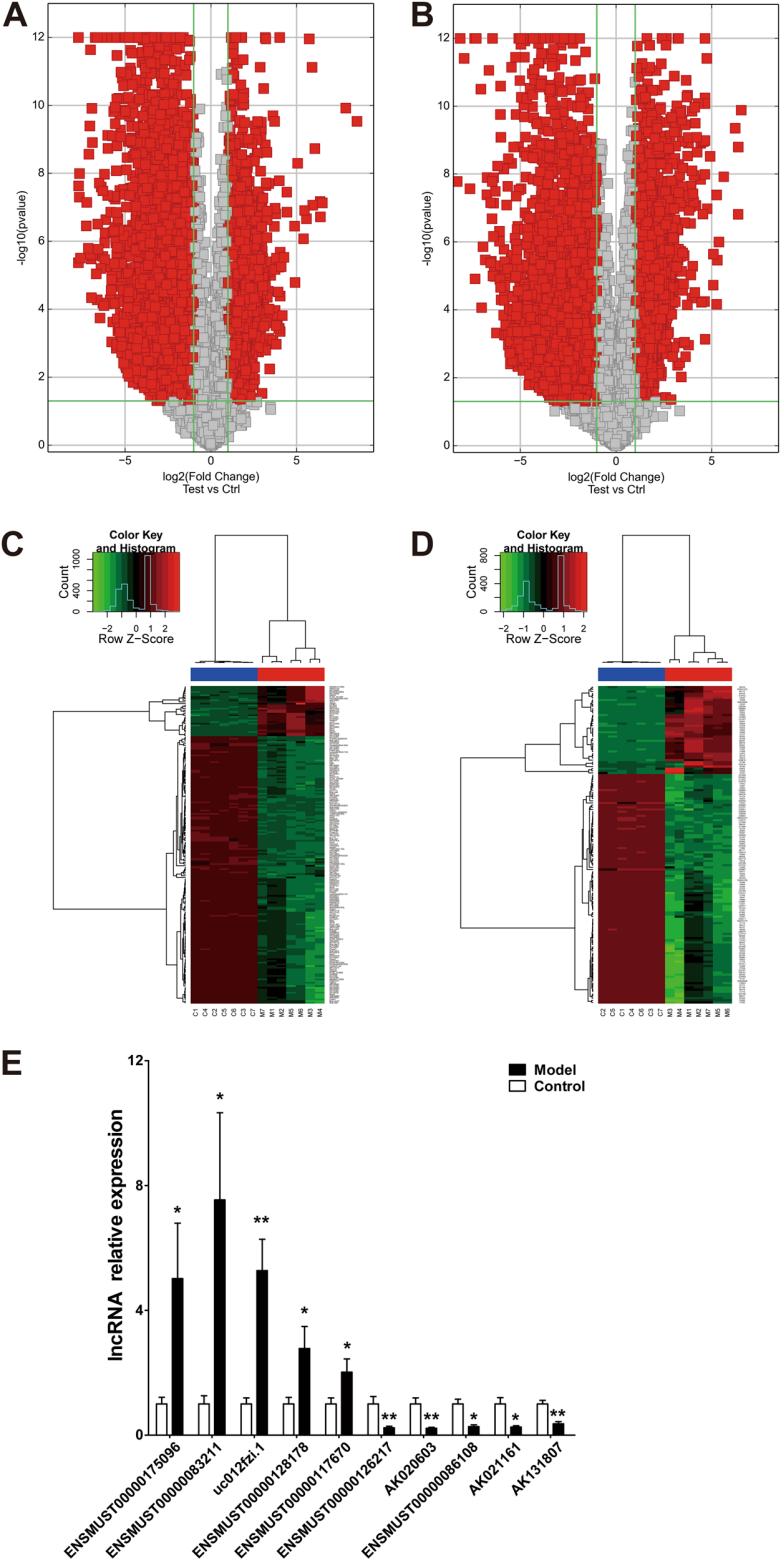
Microarray analysis for identification of differentially expressed lncRNAs and mRNAs between damaged livers and healthy controls. **a** Volcano plot of differentially expressed lncRNAs between hepatic damage models and controls. *N* = 7 for each group. **b** Volcano plot of differentially expressed mRNAs between CCl_4_ and control groups. *N* = 7 for each group. Two longitudinal green lines are fold change lines in **a** and **b**. Red symbols indicate downregulation >50% (left) or upregulation >2.0-fold change (right) in CCl_4_-treated mice relative to the control counterparts. The horizontal green line represents *P* < 0.05. Ctrl and Test represent control and CCl_4_ groups, respectively. **c** The Hierarchical clustering heatmap of differentially expressed lncRNAs between damaged livers and healthy controls. *N* = 7 for each group. **d** The Hierarchical clustering heatmap of differentially expressed mRNAs between damaged livers and healthy controls. Red color indicates high relative expression, and green indicates low relative expression. *N* = 7 for each group. C represents control group, and M represents CCl_4_ group. **e** Verification of microarray results with qRT-PCR. *N* = 3 for each group. *P < 0.05 and **P < 0.01 vs. control

We went on to characterize the expression profiles of lncRNAs and mRNAs in seven CCl_4_-treated livers and seven control livers using microarray analysis, which allows for detection of 19,023 lncRNAs and 14,824 mRNAs. The results have been stored in the Gene Expression Omnibus repository with an access number GSE68289. Our data identified 6745 upregulated and 5797 downregulated lncRNAs, out of a total of 12,542 ones detected, in CCl_4_-treated livers relative to control based upon the criteria of FC ≥2 for upregulation and ≤0.5 for downregulation and *P* < 0.05. On the other hand, 4631 upregulated and 3932 downregulated mRNAs were found out of a total of 8563 ones detected (Fig. 2a, b). Under the cutoff standard of the top 1% FC arranged from high to low, the number of differentially expressed lncRNAs was reduced to 190, of which 30 were upregulated and 160 downregulated. The number of differentially expressed mRNAs was narrowed down to 148 with 41 being upregulated and 107 downregulated. This filtering gave the most significantly deregulated lncRNAs and mRNAs in CCl_4_-induced hepatic damage (Fig. 2c, d). The lists of the differently expressed lncRNAs and mRNAs are summarized in Table 1 and Table 2, respectively.

**Table 1:** The top 10 of the results of lncRNAs with an up or downregulation in expression in the hepatic damage models compared with control

Gene symbol	Seq name	Fold change	*P* value	Reg.	RNA length (b)	Model (raw)	Control (raw)	Model (normalized)	Control (normalized)
Rpph1	ENSMUST00000175096	367.75	<0.001	Up	319	10,878.23	29.30	13.43	4.90
Vaultrc5	ENSMUST00000083211	232.90	<0.001	Up	143	26,870.34	133.39	14.85	6.98
Trp73	ENSMUST00000083211	90.27	<0.001	Up	1861	3510.71	51.71	12.19	5.69
Rprl2	NR_004439	83.38	<0.001	Up	238	837.63	7.24	9.35	2.97
Jarid2	2_00008059	82.43	<0.001	Up	327	10,653.61	127.06	13.25	6.89
Rmrp	ENSMUST00000157463	66.49	<0.001	Up	271	15,290.54	262.26	13.94	7.89
AK155692	uc029sug.1	61.14	<0.001	Up	532	13,804.82	229.16	13.63	7.70
M34473	uc009cfw.1	59.62	<0.001	Up	796	28,404.87	537.14	14.74	8.84
H2-Eb1	uc029thf.1	53.28	<0.001	Up	1416	2185.64	43.92	11.20	5.46
Gm25403	ENSMUST00000082971	47.59	<0.001	Up	143	4705.24	86.45	11.96	6.39
AK019750	AK019750	212.43	<0.001	Down	436	11.91	2207.26	2.95	10.69
AK131807	AK131807	211.89	<0.001	Down	879	208.64	16,792.93	5.73	13.46
Mup-ps15	ENSMUST00000121741	209.91	<0.001	Down	539	51.40	7254.99	4.56	12.27
AK020603	AK020603	204.43	<0.001	Down	431	61.37	7544.16	4.65	12.33
Mup-ps12	ENSMUST00000126217	202.67	<0.001	Down	549	317.35	56,757.47	7.56	15.22
humanlincRNA2050	AI507368	195.38	<0.001	Down	458	175.87	13,927.47	5.57	13.18
Gm14450	ENSMUST00000119984	154.47	<0.001	Down	240	183.91	17,550.66	6.25	13.52
Mup-ps17	ENSMUST00000120723	151.37	<0.001	Down	218	57.77	6435.20	4.87	12.11
Mup-ps21	ENSMUST00000119998	142.45	<0.001	Down	448	837.47	75,777.63	8.48	15.64
Mup-ps4	ENSMUST00000169784	139.43	<0.001	Down	474	76.74	8552.54	5.38	12.50

To identify differentially expressed lncRNAs, we performed a fold change (FC) filtering between CCl_4_ and control groups. The cutoff standard is the top 1% FC arranged from high to low. Seq name—lncRNA name. Absolute fold change—absolute fold change between the two groups. Reg—regulation in the hepatic damage model compared to control; “Up” indicates upregulated lncRNA in hepatic damage model compared with control; “Down” indicates downregulated one in hepatic damage model compared with control. Model-normal (raw)—raw intensities of each sample. Model-normal (normalized)—normalized intensities of each sample (log2-transformed). The list only shows the top 10 of the results of lncRNAs with an up or downregulation in expression in the hepatic damage models vs. control

**Table 2:** The top 10 of the results of mRNAs with an up or downregulation in expression in the hepatic damage models compared with control

Gene symbol	Seq name	Fold change	*P* value	Reg.	RNA length (b)	Model (raw)	Control (raw)	Model (normalized)	Control (normalized)
S100a9	NM_009114	93.28	<0.001	Up	488	1697.10	21.85	11.02	4.48
Cd177	NM_026862	83.11	<0.001	Up	2733	911.45	8.10	9.43	3.05
Csta	NM_001033239	82.98	<0.001	Up	2731	1008.21	5.58	9.07	2.69
Lcn2	NM_008491	74.53	<0.001	Up	853	10,2947.34	1757.61	16.61	10.39
S100a8	NM_013650	49.25	<0.001	Up	392	41,037.86	775.43	14.95	9.33
Ctsg	NM_007800	41.47	<0.001	Up	1004	3485.29	52.37	11.00	5.62
BC100530	NM_001082546	39.04	<0.001	Up	453	6273.79	87.57	11.70	6.41
Stfa1	NM_001082543	38.22	<0.001	Up	418	2755.89	27.06	10.04	4.79
Mzb1	NM_027222	38.02	<0.001	Up	928	10,548.88	221.29	12.90	7.66
S100g	NM_009789	33.85	<0.001	Up	480	1528.51	26.02	9.76	4.67
Elovl3	NM_007703	314.88	<0.001	Down	1879	16.64	4321.26	3.28	11.58
Ugt2b38	NM_133894	282.58	<0.001	Down	1894	7.64	2367.94	2.64	10.78
Hamp2	NM_183257	245.42	<0.001	Down	439	20.41	5409.34	3.94	11.88
Abhd16a	NM_178592	199.24	<0.001	Down	1945	47.44	5833.68	4.35	11.98
Cul1	NM_012042	167.74	<0.001	Down	3171	7.52	1526.85	2.82	10.21
Nr1d2	NM_011584	164.74	<0.001	Down	4640	314.41	22,652.56	6.52	13.89
Rtp3	NM_153100	158.51	<0.001	Down	2460	9.51	1343.63	2.74	10.05
Gbe1	NM_028803	133.96	<0.001	Down	2988	8.00	1267.99	2.91	9.97
Cyp8b1	NM_010012	130.48	<0.001	Down	1950	3977.65	182,298.87	9.86	16.89
Fbxw14	NM_015793	123.90	<0.001	Down	1497	120.10	7174.67	5.31	12.26

To identify differentially expressed mRNAs, we performed a fold change (FC) filtering between CCl_4_ and control groups. The cutoff standard is the top 1% FC arranged from high to low. Seq name—mRNA name. Absolute fold change—absolute fold change between the two groups. Reg—regulation in the hepatic damage model compared to control; “Up” indicates upregulated mRNA in hepatic damage model compared with control; “Down” indicates downregulated one in hepatic damage model compared with control. Model-normal (raw)—raw intensities of each sample. Model-normal (normalized)—normalized intensities of each sample (log2-transformed). The list only shows the top 10 of the results of mRNAs with an up or downregulation in expression in the hepatic damage models vs. control

Next, we verified the expression alterations of 10 lncRNAs randomly selected from the differentially expressed ones identified by our microarray analysis using qRT-PCR. As depicted in Fig. 2e, all five upregulated (ENSMUST00000175096, ENSMUST00000083211, uc012fzi.1, ENSMUST00000128178, and ENSMUST00000117670) and five downregulated lncRNAs (ENSMUST00000126217, AK020603, ENSMUST00000086108, AK021161, and AK131807) were confirmed.

### Gene ontology analysis for the potential functionalities of deregulated mRNAs

**Fig. 3:**
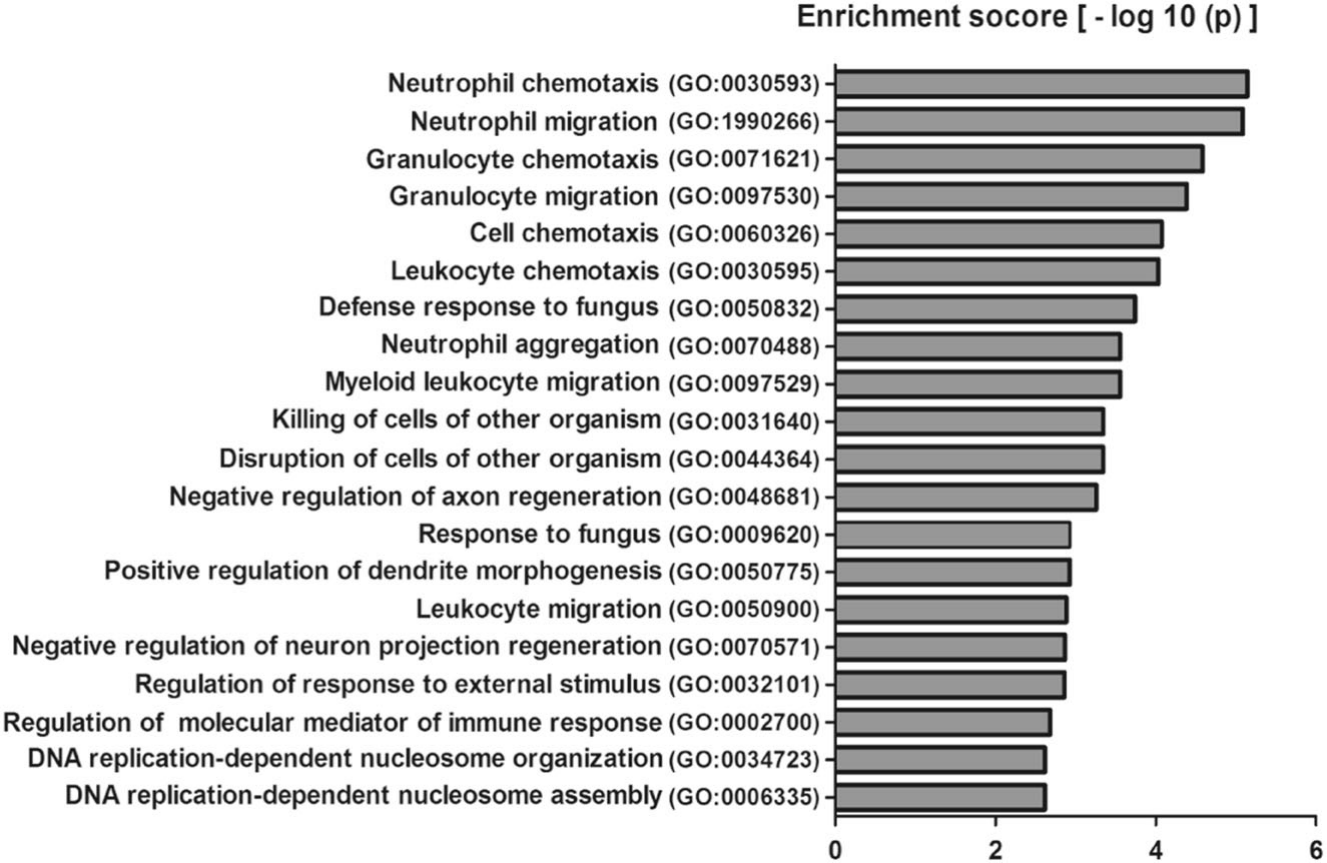
GO analysis for the potential biological functions of the differentially expressed mRNAs in hepatic damage. The chart shows the top 20 counts of the significant enrichment terms

We conducted the theoretical prediction of the functional roles of differentially expressed mRNAs by carrying out GO analysis. GO analysis derived from the GO website (www.geneontology.org) is a functional analysis that categories the functions of mRNAs into three structured networks: biological process, cellular components, and molecular function^34^. Among them, the biological process terms directly reflect the regulating functions in cells. We found that 209 biological process terms were significantly enriched with the differentially expressed mRNAs, which were strongly related to immune response, DNA replication, and regulation of MAPK, which are known to be the pivotal events in the progression of hepatic damage. Figure 3 shows the top 20 counts of the significant enrichment terms with the highest number of the differentially expressed genes.

### Analysis for lncRNA-mRNA co-expression network

**Fig. 4:**
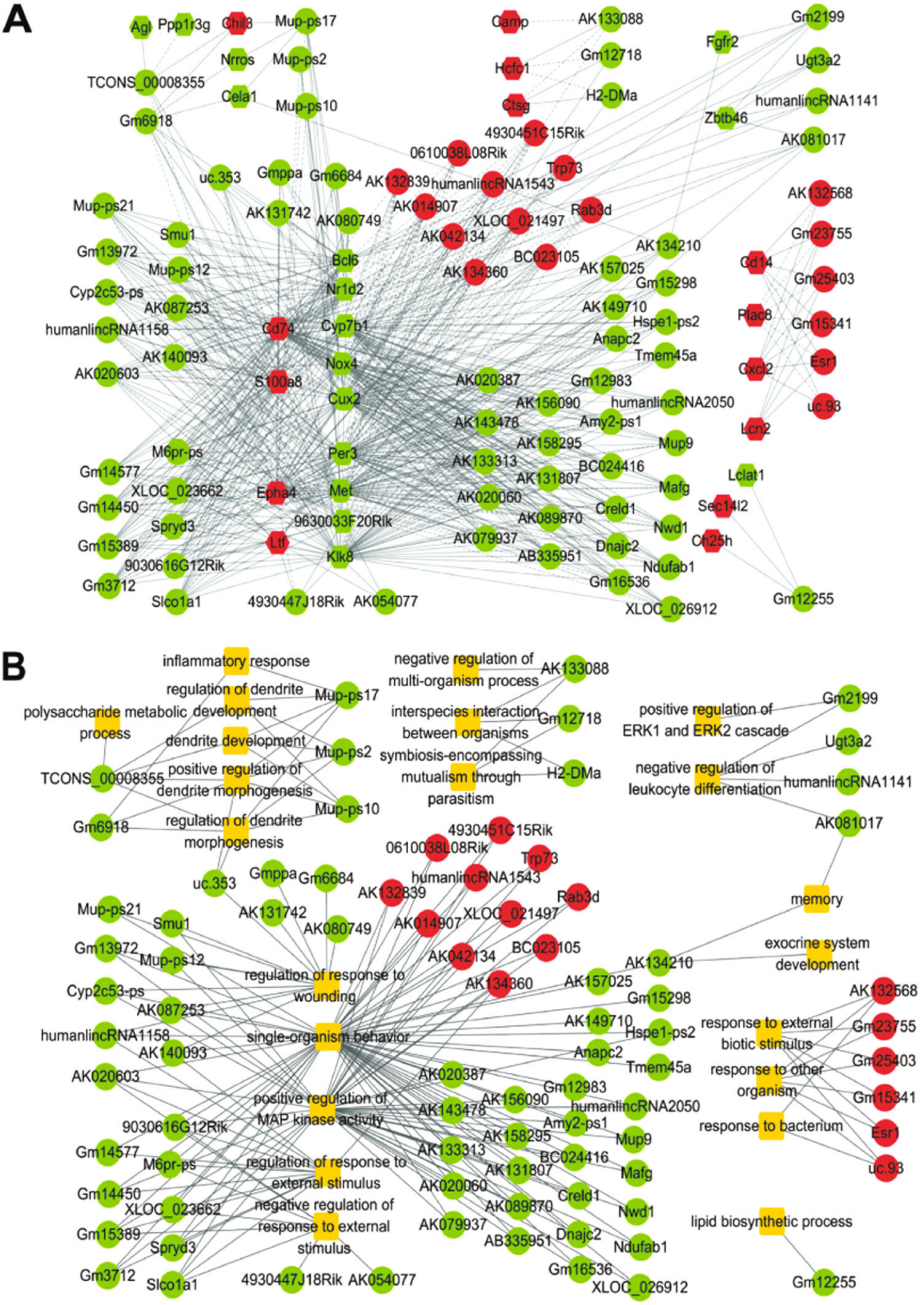
Network analysis for the potential functions of differentially expressed lncRNAs in damaged livers. **a** The co-expression network of differentially expressed lncRNAs and mRNAs. **b** The network of differentially expressed lncRNAs and biological process (BP) terms of their specific target genes. A round node represents a lncRNA, a hexagon node an mRNA, and a yellow rectangle node a BP term. Red color indicates upregulation and green downregulation. Solid lines represent positive correlation, and dash lines negative correlation. The width of the solid lines corresponds to −log10 (*p*)

Co-expression network often is used for genome-wide representation of the complex functional organization of biological systems, which is the functional annotation of unknown genes^35^. We then identified the lncRNAs that are significantly correlated with the mRNAs annotated in the enriched biological process terms and constructed the lncRNA-mRNA co-expression network with respect to hepatic damage. As illustrated in Fig. 4a, the network was composed of 84 differentially expressed lncRNAs and 30 differentially expressed mRNAs with 629 significant correlation edges (FDR <0.05).

We further investigated the potential biological functions of lncRNAs in the co-expression network by testing whether the mRNAs correlated with specific lncRNAs were significantly overlapped with the genes annotated in each biological process term. The results showed that the differentially expressed lncRNAs were involved in 22 biological processes related to hepatic damage, such as GO:1903034 for regulation of response to wounding (*P* = 0.014), GO:0032101 for regulation of response to external stimulus (*P* = 0.017), GO:0043406 for positive regulation of MAP kinase activity (*P* = 0.0207), and GO:1902106 for negative regulation of leukocyte differentiation (*P* = 0.025). These findings are displayed in Fig. 4b with the lncRNA-biological process network between the deregulated lncRNAs and the biological process terms.

### LncRNA Gm2199 is downregulated in both in vivo and in vitro models of hepatic damage

**Fig. 5:**
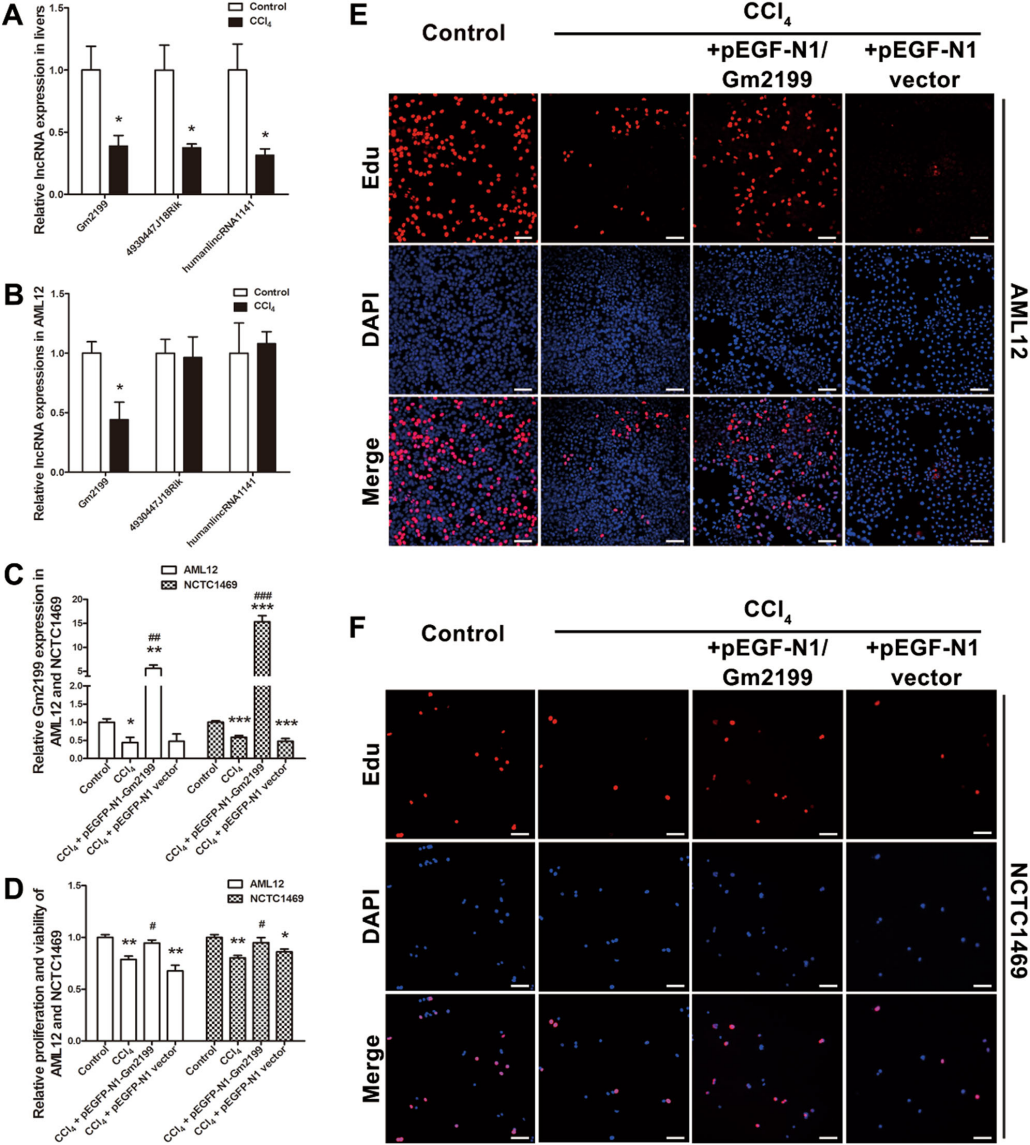
Rescuing role of lncRNA Gm2199 in hepatic damages. **a** Expression downregulation of three lncRNAs, Gm2199, 4930447J18Rik, and humanlincRNA1141, in mice with liver damages induced by CCl_4_. The lncRNAs under study were selected from the differentially expressed lncRNA in hepatic damage, which were bioinformatically predicted to be involved in the regulation of liver damage related signaling pathways. The transcript levels were determined by qRT-PCR. *N* = 5 for each group. **b** Alterations of the expression of Gm2199, 4930447J18Rik, and humanlincRNA1141 in a mouse hepatocyte line, AML12. Hepatic damage was induced by pretreating AML12 cells with 15 mmol/L CCl_4_ for 12 h. Note that only Gm2199 was found significantly downregulated in hepatic damage in vitro. *N* = 3 for each group. **c** The verification of overexpression of Gm2199 in AML12 and NCTC1469 cells transfected with pEGFP-N1-Gm2199 plasmid. *N* = 3 for each group. **d** The rescuing effect of lncRNA Gm2199 on the proliferation capacity of AML12 and NCTC1469 cells treated with CCl_4_, as measured by CCK-8 assay. *N* = 3 for each group. Note that the transfection of pEGFP-N1-Gm2199 for Gm2199 overexpression abrogated the CCl_4_-inuced diminishment of proliferation rate of AML12 cells, whereas the negative control construct failed to elicit any significant changes. The experiment data were converted to relative values over the control group and were expressed as mean ± SEM. *P < 0.05, **P < 0.01, and ***P < 0.001 vs. control; ^#^*P* < 0.05, ^##^*P* < 0.01 and ^###^*P* < 0.001 vs. CCl_4_. The rescuing effect of Gm2199 on the proliferation capacity of AML12 (**e**) and NCTC1469 (**f**), as measured by Edu staining. The scale bars represent 20 μm

To get some insight into the functional roles of the differentially expressed lncRNAs in our models, we selected Gm2199, humanlincRNA1141, and 4930447J18Rik for experimental investigations. These lncRNAs were predicted to be related to the positive regulation of ERK1/2 cascade, the negative regulation of leukocyte differentiation, and the response to external stimulus, respectively (Fig. 4b). The information about these three lncRNAs is provided in Table 3. We then observed significant downregulation of these three lncRNAs in CCl_4_-treated livers relative to control (Fig. 5a). We then further verified that Gm2199 was significantly downregulated by 0.55-fold (*P* < 0.01) in the AML12 cells treated with CCl_4_ compared with control (Fig. 5b), however the expression of humanlincRNA1141 and 4930447J18Rik remained unaltered. Thus, we decided to further investigate the relation between Gm2199 and proliferation of hepatocytes in hepatic damage. The Gm2199 sequence and conservation information are listed in Supplementary Tables S2–S4.

**Table 3:** Basic information of lncRNA Gm2199, humanlincRNA1141, and 4930447J18Rik

Gene symbol	Seq name	Type	Reg	*P* value	FDR	FC	Length	Chrom	Strand	txStart	txEnd
Gm2199	ENSMUST00000119065	Intergenic	Down	<0.001	<0.001	72.05	874	chr5	−	80900749	80901623
humanlincRNA1141	AA189272	Intergenic	Down	<0.001	<0.001	32.19	546	chr2	+	43692585	43693130
4930447J18Rik	NR_045959	Intergenic	Down	<0.001	<0.001	130.07	825	chr14	+	47898863	47944189

Reg—regulation in the hepatic damage model compared to control. FC—absolute fold change between the CCl_4_ and control groups. Chrom—the number of the chromosome where lncRNA locates. Strand—the coordinate on the genome where lncRNA start to transcribe. txEnd—the coordinate on the genome where lncRNA stop to transcribe

### LncRNA Gm2199 promotes the proliferation of damaged hepatocyte lines

One of the detrimental changes of damaged liver is diminished capability of regeneration of hepatocytes due to impaired proliferation. We therefore decide to investigate whether Gm2199 promotes hepatocyte proliferation. To this end, we overexpressed Gm2199 in hepatocyte lines, AML12 and NCTC1469, in vitro by transfection of pEGFP-N1-Gm2199 before treating the cells with CCl_4_. As illustrated in Fig. 5c, Gm2199 levels in both overexpression cell lines were robustly increased by 12.74-fold (*P* < 0.01) and 14.96-fold (*P*  < 0.001), respectively, compared to the cells transfected with pEGFP-N1 vector as a negative control. Strikingly, both of the hepatocyte lines with overexpression of Gm2199 exhibited significantly higher proliferation rate than the cells without Gm2199 overexpression measured by cell counting kit-8 (CCK-8) analysis (*P* < 0.05) (Fig. 5d) and 5-ethynyl-2′-deoxyuridine (Edu) staining (Fig. 5e, f).

### LncRNA Gm2199 protects mouse livers from injury induced by CCl_4_ in vivo

**Fig. 6:**
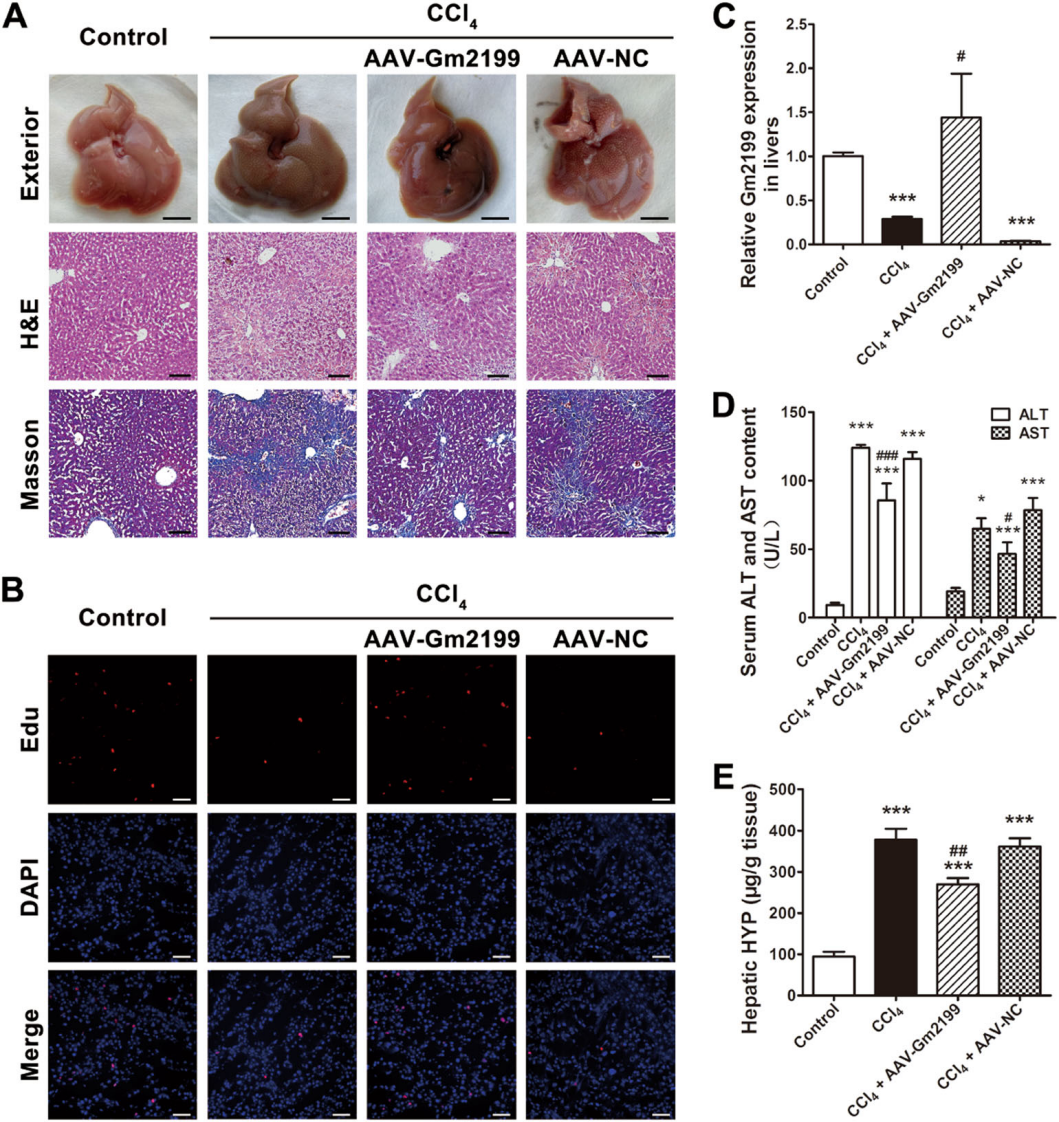
The rescuing role of Gm2199 in mouse-damaged livers. **a** The exterior and histomorphology appearances of Control, CCl_4_, CCl_4_ + AAV-Gm2199, and CCl_4_ + AAV-NC-treated livers. The scale bars of exterior represent 1 cm, and the scale bars of H&E and Masson represent 10 μm. **b** Edu staining for proliferating hepatocytes in Control, CCl_4_, CCl_4_ + AAV-Gm2199, and CCl_4_ + AAV-NC-treated livers. The scale bars represent 20 μm. **c** The relative expression of Gm2199 in Control, CCl_4_, CCl_4_ + AAV-Gm2199, and CCl_4_ + AAV-NC-treated livers, measured by qRT-PCR. The rescuing role of Gm2199 in serum ALT and AST content (**d**) and hepatic HYP (**e**) of mice treated with Control, CCl_4_, CCl_4_ + AAV-Gm2199, and CCl_4_ + AAV-NC. The experiment data were expressed as mean ± SEM (*n* = 4 for each group). *P < 0.05 and ***P < 0.001 vs. control; ^#^*P* < 0.05, ^##^*P* < 0.01, and ^###^*P* < 0.001 vs. CCl_4_

To observe whether Gm2199 could protect mouse liver from injury induced by CCl_4_, we overexpressed Gm2199 in mouse livers by injecting adeno-associated virus (AAV)-Gm2199 and AAV-negative control (NC) through tail vein 1 week earlier than the intraperitoneal injection of CCl_4_. The AAV-Gm2199 is able to particularly combine to hepatocyte genome in vivo and intracellularly overexpressed Gm2199 it carries. At the end of 3 weeks after the first injection of CCl_4_, the expression of Gm2199 in livers infected with AAV-Gm2199 was significantly higher, compared to livers treated with CCl_4_ or CCl_4_ + AAV-NC (*P* < 0.05) (Fig. 6c). The livers overexpressing Gm2199 showed more regular and living hepatocytes with less inflammatory infiltration and collagen deposition in ECM (Fig. 6a). Edu staining also showed that livers treated with CCl_4_ + AAV-Gm2199 contained more proliferating hepatocytes than livers treated with CCl_4_ or CCl_4_ + AAV-NC (Fig. 6b). Moreover, the serum ALT and AST (Fig. 6d) and hepatic HYP (Fig. 6e) of mice treated with CCl_4_ + AAV-Gm2199 were significantly lower compared with that of mice injected with CCl_4_ or CCl_4_ + AAV-NC. Those results showed that Gm2199 is able to protect mouse livers from injury induced by CCl_4_ in vivo.

### LncRNA Gm2199 promotes the activation and the expression of ERK1/2 in damaged hepatocyte lines

**Fig. 7:**
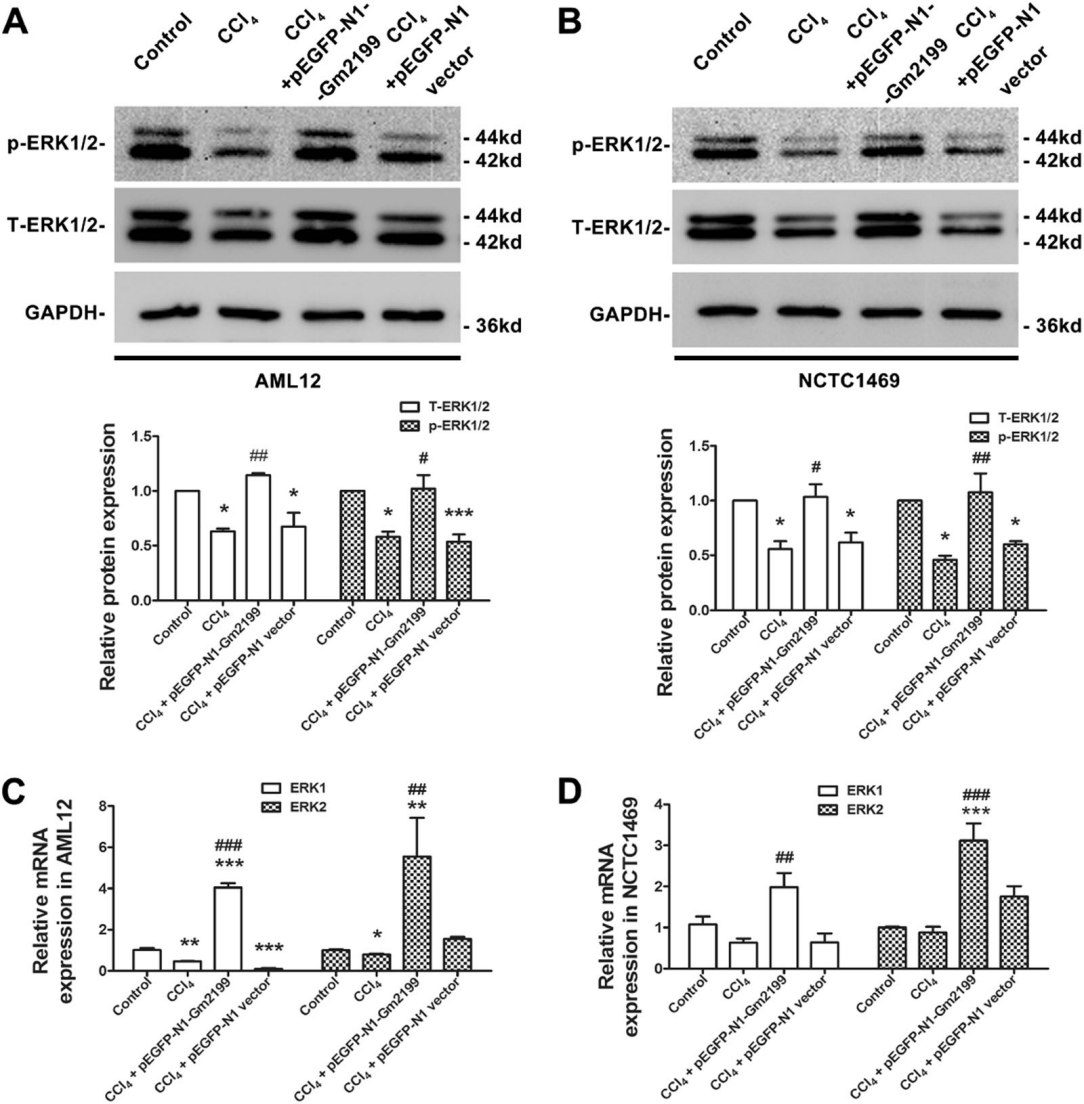
The effects of Gm2199 on the activation and the expression of ERK1/2 in damaged hepatocytes. Representative western blot analysis results for p-ERK1/2 and T-ERK1/2 protein expression from Control, CCl_4_, CCl_4_ + pEGFP-N1-Gm2199, and CCl_4_ + pEGFP-N1 vector-treated AML12 (**a**) and NCTC1469 (**b**). QRT-PCR analysis results for ERK1 and ERK2 mRNA expression from Control, CCl_4_, CCl_4_ + pEGFP-N1-Gm2199, and CCl_4_ + pEGFP-N1 vector-treated AML12 (**c**) and NCTC1469 (**d**). The experiment data were converted to relative values over the control group and were expressed as mean ± SEM (*n* = 3 for each group). *P < 0.05, **P < 0.01, and ***P < 0.001 vs. control; ^#^*P* < 0.05, ^##^*P* < 0.01, and ^###^*P* < 0.001 vs. CCl_4_

As Gm2199 was predicted to be related with the positive regulation of ERK1/2 cascade (Fig. 4b), which was proved to be related to the proliferation of hepatocyte^24,29^, we investigated whether overexpression of Gm2199 could promote the expression level of ERK1/2 in damaged hepatocytes. As illustrated in Fig. 7a, the expressions of phosphorylate-ERK1/2 (p-ERK1/2) and total-ERK1/2 (T-ERK1/2) were both significantly decreased in AML12 treated with CCl_4_ compared with control (*P* < 0.05). Whereas, the overexpression of Gm2199 reversed the phenomenon in AML12 treated with CCl_4_. A similar effect of Gm2199 was also observed in NCTC1469 (Fig. 7b). To further investigate the relation between Gm2199 overexpression and the expression of ERK1/2 in damaged hepatocytes, we detected ERK1/2 mRNA levels in AML12 and NCTC1469 with qRT-PCR. As shown in Fig. 7c, d, ERK1/2 mRNA expressions were robustly elevated in CCl_4_ + pEGFP-N1-Gm2199-treated AML12 and NCTC1469 relative to CCl_4_-treated cells.

### Overexpression of Gm2199 promoted the proliferation of normal AML12 and NCTC1469 through upregulation of p-ERK1/2

**Fig. 8:**
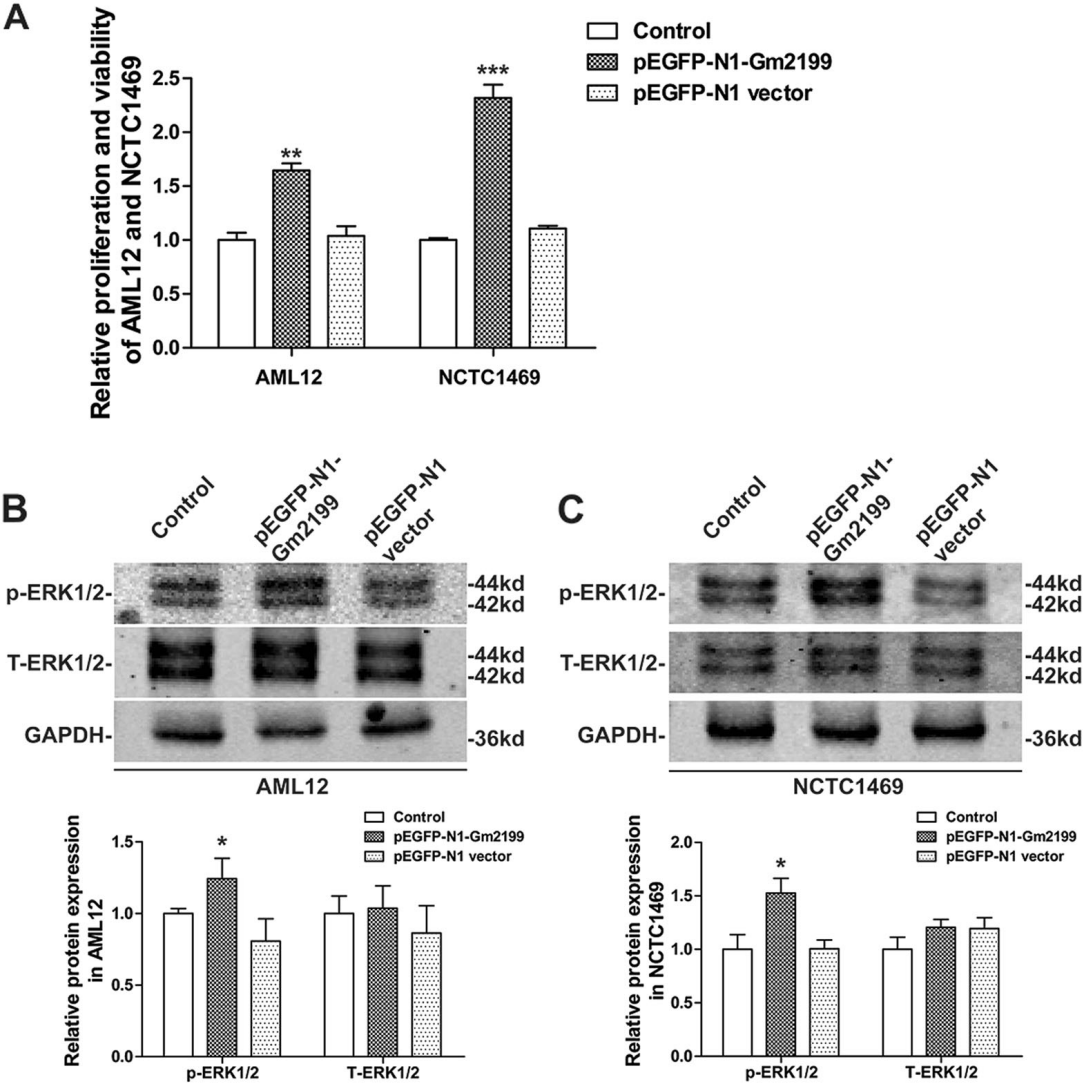
The effects of overexpression of Gm2199 on the the proliferation and viability and the expression of ERK1/2 in normal AML12 and NCTC1469. PEGFP-N1-Gm2199 was transfected into normal AML12 and NCTC1469 to overexpress Gm2199, and pEGFP-N1 was transfected into normal AML12 and NCTC 1469 as negative control. **a** Relative cellular proliferation and viability of AML12 and NCTC1469 determined by CCK-8 assay. Representative Western blot and relative brand intensity analysis results for p-ERK1/2 and T-ERK1/2 proteins in AML12 (**b**) and NCTC1469 (**c**). The experiment data were converted to relative values over the control group and were expressed as mean ± SEM (*n* = 3 for each group). *P < 0.05, **P < 0.01, and ***P < 0.001 vs. pEGFP-N1 group

To explore whether overexpression of Gm2199 affects the proliferation and viability of AML12 and NCTC1469 without CCl_4_ treatment, we transfected pEGFP-N1-Gm2199 into the normal cells to overexpress Gm2199. pEGFP-N1 vector was also transfected into normal cells as negative control. The cellular proliferation was determined by CCK-8 after 36 h of the transfections. As shown in Fig. 8a, pEGFP-N1-Gm2199 significantly increased the proliferation and viability of AML12 and NCTC1469, compared with pEGFP-N1 vector group. To further confirm whether overexpression of Gm2199 affects the level of ERK1/2 in normal AML12 and NCTC1469, the protein levels of ERK1/2 were determined by western blot after 24 h of the transfections. As shown in Fig. 8b, c, pEGFP-N1-Gm2199 significantly increased the levels of p-ERK1/2 in AML12 and NCTC1469, compared with pEGFP-N1 vector group. However, the levels of T-ERK1/2 in AML12 and NCTC1469 showed no significant difference between each group. These results suggested that overexpression of Gm2199 in normal AML12 and NCTC1469 promoted cellular proliferation and viability by increasing the level of p-ERK1/2.

## Discussion

In this study, we have identified deregulated lncRNAs in a mouse model of liver damage using microarray and qRT-PCR. Theoretical analyses allowed us to have acquired some preliminary messages about the possible involvement of the deregulated lncRNAs in regulating signaling pathways known to be important to the pathogenesis of hepatic damage. In particular, our data unraveled that a lncRNA, Gm2199, was markedly downregulated in both in vivo mouse model of liver damage and in vitro cellular model of hepatocyte damage. The downregulation of Gm2199 might contribute to the hepatic damage manifested by diminished proliferation capacity of hepatocytes. Supplement of Gm2199 by forced expression to damaged AML12 and NCTC1469 hepatocytes effectively restored the decreased capacity of hepatocyte proliferation. Moreover, Gm2199 overexpression in vivo protected liver injury from CCl_4_. The fact not only indicates Gm2199 as an anti-liver damage lncRNA but also opens up a new opportunity for further investigations on the possibility of it as a therapeutic target for the treatment of liver damage.

LncRNAs have been demonstrated the possible usefulness for diagnosing and treating hepatic diseases^14,15,36^. MEG3 and GAS5 were found to inhibit liver fibrosis^16,18^. TUG1 was showed to protect against cold-induced injury of mouse livers^37^. However, the panoramic view on what and how lncRNAs contribute to hepatic damages remained unclear. In conjunction with the expression profiling of lncRNAs and mRNAs, we were able to sort out a subset of deregulated lncRNAs and mRNAs that together constitute a co-expression network bearing significant implications in the signaling pathways relevant to liver damages. Among the various biological process terms, immune response, DNA replication, and regulation of MAPK are known to be closely related to liver damages. It has been documented that immune response, recruiting immune cells such as leukocytes and Natural killer cells, which attack hepatocytes and activate HSCs for proliferating, could accelerate the development of hepatic damage^38^. On the other hand, DNA replication is the first step and a key determinant of the proliferation of hepatocytes and HSCs and is critically regulated by the MAPK signal pathway^39^.

Of those 84 lncRNAs implicated in liver damages, AK134360 is the only one that was found to be upregulated and related to the regulation of responses to external stimuli and of MAPK activity in hepatic damage. The MAPK pathway, mediating external stimulus, is a known intermediate in both fibrogenic and inflammatory regulatory cascades^40–43^. Therefore, AK134360 may contribute to the development of hepatic damage, which needs our further research to be confirmed. In contrast, 4930447J18Rik and AK054077 downregulated in hepatic damage, were showed only related to negative regulation of response to external stimulus. As a result, the loss of those lncRNAs may cause the disbalance of the regulating processes like immune response, aggravating the hepatic damage progress. Moreover, humanlincRNA1141 and Ugt3a2 downregulated in hepatic damage, were showed only related to negative regulation of leukocyte differentiation. As leukocyte differentiation was proved to promote hepatic inflammation and fibrosis progression^44,45^, those kinds of lncRNAs may inhibit inflammation to attenuate hepatic damage. So, it is worthy to further overexpress these downregulated lncRNAs in mouse livers to try to inhibit hepatic damage in future researches.

Normally, different cell type in a liver expresses different level of genes including non-coding RNAs^46^. MicroRNA-706 is downregulated in hepatocytes but unchanged in non-parenchymal cells under oxidative stress^47^. LncRNA MEG3 and GAS5 remarkably decrease in livers and HSCs but not hepatocytes, in response to fibrotic stimulation^16,18^. In this respect, we found that 4930447J18Rik and humanlincRNA1141 were deregulated in damaged livers but unchanged in damaged hepatocytes. Maybe, one of the reasons of the phenomenon was that the lncRNAs were mainly downregulated in other cell types except for the hepatocytes under the damaged condition.

Our results showed that the proliferation of hepatocytes was significantly inhibited by chronic CCl_4_ administration for 24 h in vitro and for 3 weeks in vivo. Although some studies showed that the proliferation of hepatocytes in vivo increased right after CCl_4_ administration^48,49^, others showed that the proliferation of hepatocytes in vivo was inhibited after several times and weeks of CCl_4_ administration^50,51^. A possible reason of the contradiction results maybe the difference between the duration of CCl_4_ administration. Similar with our detection, Jung et al.^50^ also found that CCl_4_ notably decreased hepatocyte proliferation in vitro. The mechanism of the inhibition is attributed to the decrease of the expression of ERK1/2 in hepatocytes, as proved in our results. Therefore, partially through the inhibition of ERK1/2 pathway, the chronic administration of CCl_4_ inhibits the proliferation of hepatocytes in vitro and in vivo.

On the other hand, the proliferation of hepatocytes can be promoted by interventions including gene therapies to treat liver injury^52^. Xu et al.^36^ found that lncRNA LALR1 was associated with liver regeneration, and overexpression of it accelerated hepatocyte proliferation to promote liver regeneration. We overexpressed Gm2199 in damaged hepatocyte lines in vitro and livers in vivo, to make it clear that whether it could inhibit hepatic damage by promoting the proliferation of damaged hepatocytes. Inspiringly, overexpression of Gm2199 notably increased the proliferation of damaged hepatocytes and attenuated liver damages compared with CCl_4_-treated groups. Those results indicate that lncRNA Gm2199 has ability of protecting hepatocytes from injury and promote damaged liver regeneration by promoting hepatocyte proliferation.

The activation or increase of ERK1/2 is able to promote hepatocyte proliferation and viability^53^. Murata et al.^54^ found that the activation of ERK1/2 pathways promoted liver regeneration after hepatectomy in mice. Coutant et al.^26^ proved that ERK1/2 activation supports both proliferation and viability of normal hepatocytes. In our research, Gm2199 was the only one predicted to be related with positive regulation of ERK1/2. Gm2199 was also downregulated both in damaged livers and damaged hepatocytes, and the expression and the activation of ERK1/2 were inhibited in damaged hepatocytes. The results suggested that the loss of the positive regulation of Gm2199 in damaged hepatocytes contributed into the inhibition of proliferation and the decrease of ERK1/2. Our farther results proved that overexpression of Gm2199 increased T-ERK1/2 and p-ERK1/2 protein levels in damaged hepatocytes, which can be attributed to the elevated ERK1/2 mRNA expression. However, the reason of upregulated ERK1/2 mRNA induced by overexpression of Gm2199 needs further investigation. As a conclusion, overexpression of Gm2199 is able to promote damaged hepatocyte proliferation through upregulation of ERK1/2.

In conclusion, we have characterized the expression profiles of lncRNAs and mRNAs and identified the deregulated lncRNAs and mRNAs in the setting of liver damages induced by CCl_4_. We obtained the functional implications of 84 lncRNAs and 30 mRNAs in the regulation of hepatic damages. These findings should lay the groundwork for further investigations into the nature and mechanisms of these lncRNAs for their possible involvement in hepatic damage and maybe other types of liver disease as well. Specifically, we discovered that lncRNA Gm2199 was markedly downregulated in damaged livers and hepatocytes, and this abnormal downregulation might be one of the factors leading to impaired hepatocyte proliferation because Gm2199 replacement by overexpression was able to rescue the reduced proliferation capacity and ERK1/2 expression of damaged hepatocytes.

## Electronic supplementary material

Supplementary Table S1

Supplementary Table S2

Supplementary Table S3

Supplementary Table S4
