# Supplementary material for: Long non-coding RNA Gm2199 rescues liver injury and promotes hepatocyte proliferation through the upregulation of ERK1/2
Source: Cell Death Dis. 2018 May 22;9(6):602. doi: 10.1038/s41419-018-0595-9 (PMC5964236; doi:10.1038/s41419-018-0595-9)
Supplement: Supplementary file 1 — Supplementary Table S1 [file 41419_2018_595_MOESM1_ESM.docx]

**Supplementary Table S1 Primers of lncRNAs and mRNAs in Mouse Livers.**

| Primer | Forward | Reverse |
| --- | --- | --- |
| ENSMUST00000175096 | GCAGACTGGGCAGGAGAAGC | CACCTCAGCCATTGAACTCG |
| ENSMUST00000083211 | CACAACCAACCACTTACCTA | GTCTCGAACCAAACACTCAC |
| uc012fzi.1 | TTTCCTCAAACTCACTCCTA | CCCTGAAAATACTCCACGCA |
| ENSMUST00000128178 | CAGGAACCCACTACCATACA | AAAGGGCTCAGACTCAAGAC |
| ENSMUST00000117670 | CTTCCGATACAACGGGCTAA | ACCACAGGTTTCTGGCTTCA |
| ENSMUST00000126217 | AATGAATGGGGAAACCTTCC | CATTCTAGGGCCTGGAGACA |
| AK020603 | CTGGTTTGGAACTCCAGAGG | CAGGCCTTGAGCATAAGAGG |
| ENSMUST00000086108 | CCTTTTTCGTTATGCCAAGC | CCAAGTTAGCAGTGCCATCA |
| AK021161 | CATCCGTTACCTGCATGATG | CCTCCTTGGCTGAGTGTCTC |
| AK131807 | GTAGCAATGCTGCTGGTGAG | CCCTCTTCCCCGACTATAGG |
| Gm2199 | TACACATCCCTTCCACGTGA | ATGCCTTCTTCCCGGTAGAT |
| humanlincRNA1141 | CCAGAAGACCCTGGATGTAC | ATTGAGTACCGCATGGGTTT |
| 4930447J18Rik | CAGGGCTACCCTGACACTTC | CCCCATATCCAGTGCAGACT |
| Col 1α1 | CCTGGCAAAGACGGACTCAAC | GCTGAAGTCATAACCGCCACTG |
| αSMA | TCCCTGGAGAAGAGCTACGAACT | AAGCGTTCGTTTCCAATGGT |
| TIMP-1 | GCATGGACATTTATTCTCCACTGT | TCTCTAGGAGCCCCGATCTG |
| MMP-2 | CCCTCAAGAAGATGCAGAAGTTC | TCTTGGCTTCCGCATGGT |
| TNF-α | GTGGAACTGGCAGAAGAG | AATGAGAAGAGGCTGAGAC |
| TGF-β1 | GCCTGAGTGGCTGTCTTTTGA | GCTGAATCGAAAGCCCTGTATT |
| ERK1 | TCCAAGGGCTACACCAAATC | AGGTAGTTTCGGGCCTTCAT |
| ERK2 | CACCAACCATTGAGCAAATG | GTTCAGCAGGAGGTTGGAAG |
| GAPDH | TGCACCACCAACTGCTTAG | GGATGCAGGGATGATGTTC |

Col1α1, collagenⅠα1; αSMA, α smooth muscle actin; TIMP-1, tissue inhibitors of matrix metalloproteinases-1; MMP-2, matrix metalloproteinases-2; TNF-α, tumor necrosis factor-α; TGF-β1, transforming growth factor-β1; ERK1, extracellular signal regulated kinase 1; ERK2, extracellular signal regulated kinase 2; GAPDH, glyceraldehyde-3-phosphate dehydrogenase.
