# Supplementary material for: Long non-coding RNA Gm2199 rescues liver injury and promotes hepatocyte proliferation through the upregulation of ERK1/2
Source: Cell Death Dis. 2018 May 22;9(6):602. doi: 10.1038/s41419-018-0595-9 (PMC5964236; doi:10.1038/s41419-018-0595-9)
Supplement: Supplementary file 2 — Supplementary Table S2 [file 41419_2018_595_MOESM2_ESM.docx]

**Supplementary Table S2 The FASTA sequence of mouse lncRNA Gm2199 transcript**

| **Locus** | **Sequence** |
| --- | --- |
| >5 dna:chromosome chromosome:GRCm38:5:80900750:80901623:-1 | GGACTCACCATCCTGTCCCAGCCGCTCATATATGTGAAAGTGCTCATCCAGGTGGGATATGAACCTCTTCCTCCAACAATAGGACAGAATATTTTTGGGAGACAAGTATGTCAGCTTCCTGGCCTCTTTTGCTATGCTCAGCACATTGCAAGCATCGATGGGAGGCGTGGGTTGTTCACAGGCTTGACTCCAAGACTGTGCTCAGGAGTCCTTGGAACTGTGGTCTATGGGAAAGTCTTACAGTATTACCAGGAGTCTGAGAAACCTAAGGAGTTAGGATCTGTAACTGTACAAAAAGAATGTTCATCCTCCTTTGACTGAGTTATCAAAGAGACAACCTGAGAGATGATTGCTCATTCTGCTGCTACCCTCATTACACATCCCTTCCACGTGATCACTCTGAGGTCCATGGTACAGTTTATTGGCAGAGAATCTAAGTACTGTAGATTGTGTGACTCCATAGTAACCATCTACCGGGAAGAAGGCATGGTAGGATTTTTTTGTGGGTCTCATTCCTCACCTCCTAGGTGACATTTCTTTGTGGCTGTGTAACTCACTGGCCTATCTCATCAATACCTATGCACTGGACAGTGGGGTTTCTACCATGAATGAAATGAAAAGTTATTCCCAAGCTGTCACAGGATTCTTTGCCAGTATGTTGACATATCCCTTTGTGCTTATATCTAATCTTATGGCCATCAACAACTGTGGGCTTGCTGGTGGATCTCCTCCTTATTCCCCAATATACACTTCTTGGATAGATTGCTGGTGCATGCAACAAAAAGCAGGAAATAAGAGCCAAGGAAACAGCTTGTTTTTCCAGAAGGTTCCTTGTGGGAAAACTTACTGTTATGACCTAAGACTATTAATCTGA |
