# Supplementary material for: Long non-coding RNA Gm2199 rescues liver injury and promotes hepatocyte proliferation through the upregulation of ERK1/2
Source: Cell Death Dis. 2018 May 22;9(6):602. doi: 10.1038/s41419-018-0595-9 (PMC5964236; doi:10.1038/s41419-018-0595-9)
Supplement: Supplementary file 3 — Supplementary Table S3 [file 41419_2018_595_MOESM3_ESM.docx]

**Supplementary Table S3 The BLAST result of lncRNA Gm2199 in mouse genome and transcript databases of NCBI.**

| **Description** | **Total Score** | **Query cover** | **E value** | **Ident** | **Accession** |
| --- | --- | --- | --- | --- | --- |
| **Transcripts** | | | | | |
| Mus musculus mitochondrial carrier 2 (Mtch2), transcript variant 1, mRNA | 1415 | 100% | 0 | 96% | [NM_001317241.1](https://www.ncbi.nlm.nih.gov/nucleotide/NM_001317241.1?report=genbank&log$=nucltop&blast_rank=1&RID=CS7CZC9901R) |
| Mus musculus mitochondrial carrier 2 (Mtch2), transcript variant 2, mRNA | 1361 | 100% | 0 | 93% | [NM_001317242.1](https://www.ncbi.nlm.nih.gov/nucleotide/NM_001317242.1?report=genbank&log$=nucltop&blast_rank=3&RID=CS7CZC9901R) |
| Mus musculus mitochondrial carrier 2 (Mtch2), transcript variant 3, mRNA | 1317 | 100% | 0 | 93% | [NM_001317243.1](https://www.ncbi.nlm.nih.gov/nucleotide/NM_001317243.1?report=genbank&log$=nucltop&blast_rank=5&RID=CS7CZC9901R) |
| Mus musculus mitochondrial carrier 2 (Mtch2), transcript variant 4, mRNA | 1292 | 100% | 0 | 91% | [NM_001317244.1](https://www.ncbi.nlm.nih.gov/nucleotide/NM_001317244.1?report=genbank&log$=nucltop&blast_rank=6&RID=CS7CZC9901R) |
| **Genomic sequences** | | | | | |
| Mus musculus strain C57BL/6J chromosome 5, GRCm38.p4 C57BL/6J | 1577 | 100% | 0 | 100% | [NC_000071.6](https://www.ncbi.nlm.nih.gov/nucleotide/NC_000071.6?report=genbank&log$=nucltop&blast_rank=8&RID=CS7CZC9901R) |
| Mus musculus strain C57BL/6J chromosome 2, GRCm38.p4 C57BL/6J | 187 | 96% | 1.00E-44 | 98% | [NC_000068.7](https://www.ncbi.nlm.nih.gov/nucleotide/NC_000068.7?report=genbank&log$=nucltop&blast_rank=9&RID=CS7CZC9901R) |
| Mus musculus strain C57BL/6J chromosome 12, GRCm38.p4 C57BL/6J | 87.8 | 11% | 1.00E-44 | 80% | [NC_000078.6](https://www.ncbi.nlm.nih.gov/nucleotide/NC_000078.6?report=genbank&log$=nucltop&blast_rank=10&RID=CS7CZC9901R) |

The table columns provide the following information: the description/title of matched database sequence; the total alignment scores (Total score) from all alignment segments; the percentage of query covered by alignment to the database sequence; the best (lowest) Expect value (E value) of all alignments from that database sequence; the highest percent identity (Max ident) of all query-subject alignments, and the Accession of the matched database sequence.
