# Supplementary material for: Long non-coding RNA Gm2199 rescues liver injury and promotes hepatocyte proliferation through the upregulation of ERK1/2
Source: Cell Death Dis. 2018 May 22;9(6):602. doi: 10.1038/s41419-018-0595-9 (PMC5964236; doi:10.1038/s41419-018-0595-9)
Supplement: Supplementary file 4 — Supplementary Table S4 [file 41419_2018_595_MOESM4_ESM.docx]

**Supplementary Table S4 The BLAST result of lncRNA Gm2199 in homo sapiens genome and transcript databases of NCBI.**

| **Description** | **Total Score** | **Query cover** | **E value** | **Ident** | **Accession** |
| --- | --- | --- | --- | --- | --- |
| **Transcripts** | | | | | |
| Homo sapiens mitochondrial carrier 2 (MTCH2), transcript variant 1, mRNA | 1081 | 100% | 0 | 87% | [NM_001317231.1](https://www.ncbi.nlm.nih.gov/nucleotide/NM_001317231.1?report=genbank&log$=nucltop&blast_rank=1&RID=CS7WV723014) |
| Homo sapiens mitochondrial carrier 2 (MTCH2), transcript variant 2, mRNA | 1041 | 100% | 0 | 85% | [NM_001317232.1](https://www.ncbi.nlm.nih.gov/nucleotide/NM_001317232.1?report=genbank&log$=nucltop&blast_rank=3&RID=CS7WV723014) |
| Homo sapiens mitochondrial carrier 2 (MTCH2), transcript variant 3, mRNA | 1005 | 93% | 2.00E-165 | 87% | [NM_001317233.1](https://www.ncbi.nlm.nih.gov/nucleotide/NM_001317233.1?report=genbank&log$=nucltop&blast_rank=10&RID=CS7WV723014) |
| **Genomic sequences** | | | | | |
| Homo sapiens chromosome 12, GRCh38.p7 Primary Assembly | 756 | 90% | 0 | 82% | [NC_000012.12](https://www.ncbi.nlm.nih.gov/nucleotide/NC_000012.12?report=genbank&log$=nucltop&blast_rank=12&RID=CS7WV723014) |
| Homo sapiens chromosome 16, GRCh38.p7 Primary Assembly | 1268 | 99% | 2.00E-180 | 77% | [NC_000016.10](https://www.ncbi.nlm.nih.gov/nucleotide/NC_000016.10?report=genbank&log$=nucltop&blast_rank=14&RID=CS7WV723014) |
| Homo sapiens chromosome 3, GRCh38.p7 Primary Assembly | 479 | 63% | 3.00E-132 | 79% | [NC_000003.12](https://www.ncbi.nlm.nih.gov/nucleotide/NC_000003.12?report=genbank&log$=nucltop&blast_rank=16&RID=CS7WV723014) |
| Homo sapiens chromosome 11, GRCh38.p7 Primary Assembly | 997 | 82% | 2.00E-34 | 92% | [NC_000011.10](https://www.ncbi.nlm.nih.gov/nucleotide/NC_000011.10?report=genbank&log$=nucltop&blast_rank=18&RID=CS7WV723014) |

The table columns provide the following information: the description/title of matched database sequence; the total alignment scores (Total score) from all alignment segments; the percentage of query covered by alignment to the database sequence; the best (lowest) Expect value (E value) of all alignments from that database sequence; the highest percent identity (Max ident) of all query-subject alignments, and the Accession of the matched database sequence.
